# The Effect of a Total Fishmeal Replacement by *Arthrospira platensis* on the Microbiome of African Catfish (*Clarias gariepinus*)

**DOI:** 10.3390/life11060558

**Published:** 2021-06-14

**Authors:** Simon Rosenau, Elisa Oertel, Alexander Charles Mott, Jens Tetens

**Affiliations:** 1 Department of Animal Science, Georg-August-University of Göttingen, 37077 Göttingen, Germany; elisa.oertel@uni-goettingen.de (E.O.); alexandercharles.mott@uni-goettingen.de (A.C.M.); jens.tetens@uni-goettingen.de (J.T.); 2 Center for Integrated Breeding Research, Georg-August-University of Göttingen, 37075 Göttingen, Germany

**Keywords:** microbiome, microalgae, cyanobacteria, *Arthrospira platensis*, *Clarias gariepinus*, 16S rRNA, bacteria

## Abstract

An increasing number of fishmeal supplements are becoming the focus of aquaculture research, with a special emphasis on microalgae/cyanobacteria such as spirulina being considered as sustainable alternatives. New feed ingredients can have a far-reaching impact on the intestinal microbiome and therefore play an important role in the development and the health of fish. However, the influence of these alternatives on the microbiome is largely unknown. We undertook a 10 weeks feeding experiment on 120 African catfish with an initial body weight of 50.1 ± 2.95 g. To understand the effect of the spirulina supplementation, two isoenergetic experimental diets were formulated, containing either fishmeal or spirulina as a protein source. The 16S rRNA sequencing was used to analyze the intestinal bacteria microbiota. Results show that the observed richness indicated no significant statistical difference, but Chao1, ACE, Shannon, and Simpson indices indicate a possible increase in bacterial richness for the spirulina diet. The most abundant bacteria in both experimental groups were *Fusobacteriia* with the only taxa from the genus *Cetobacterium*. The bacterium from genus *Romboutsia* was more likely to be found in the microbiome of fish fed the fishmeal diet. In spirulina-fed fish, the genera *Plesiomonas* and *Bacteroides* were the most dominant microbes observed. Even though some genera were more abundant in the spirulina group, the overall microbial community structure was not affected by diets.

## 1. Introduction

The production of fish for human consumption through aquaculture is increasing steadily [1], and an increasing demand for fishmeal is predicted to continue [2]. As a result, the global marine fish stocks became strongly overfished [1] with rising concerns for the ecosystem and the future of fish populations [3]. Since the 1950s, the search for alternative protein sources is a major focus of aquafeed research [4]. However, due to the expected rise in pricing and availability, this is now a priority, as the operational costs for aquafeed are between 50–70% [5]. Current trends in aquafeed production are now more focused on the exploitation of plant ingredients, simultaneously decreasing fishmeal and oil [6]. New feed ingredients such as microalgae and cyanobacteria (including multiple species such as *Arthrospira*, *Schizochytrium*, *Tetraselmis*, etc.) are suggested as potentially cost-effective and sustainable substitute [7] and increasingly gaining importance as feed-stuff [8], but their impact on the fish gut is yet to be investigated.

The cyanobacteria *Arthrospira platensis* and *A. maxima*, known collectively as spirulina, are photosynthetic, filamentous, and spiral-shaped cyanobacteria of 0.5 mm in length [4]. It contains high proportions of protein, between 59–65% [9], and it is known that low level inclusions can boost growth performance and feed conversion ratio in African catfish [10,11]. Spirulina supplementation also contribute to increase the carotenoid content in the fish muscle and has an immune stimulating effect [10]. In other species, a positive effect on the fatty acid composition was observed, resulting in enhanced polyunsaturated fatty acids [12,13,14].

It is not only the performance of the fish that is affected by the diet but also the whole gastrointestinal tract and its microbiome [15,16,17,18,19,20,21]. Souza et al. [22] studied the microbiome of Nile tilapia fed with low concentrations of unicellular microalgae, indicating an influence of microalgae on the microbial community due to an alteration in the bacterial abundance. In support of this, Cerezuela et al. [23] showed that diets containing unicellular alga altered the intestinal microbiota and decreased the bacterial diversity in gilthead seabream (*Sparus aurata*). In contrast, Lyons et al. [24] found a greater microbial diversity in rainbow trout (*Oncorhynchus mykiss*) fed with 5% microalgae meal. However, there are a number of other factors, such as environment [25,26,27,28], season [29,30], geographic location [31,32], and host genetics [27], influencing the microbiome. The health of the gastrointestinal tract is of particular importance for host health, because it is considered to be one of the main routes of infection in fish [33]. Nevertheless, the microbiome plays an important role in host immune system and fish nutrition [34,35], which are two of the most important factors for a successful aquaculture production. For this reason, research focuses on microbiome modulating effects of feed ingredients and additives [34].

Since limited information is available about the effect of microalgae on the microbiome, the objective of this study was to investigate the effect spirulina (obtained from the cyanobacterium *Arthrospira platensis*) has on the microbiome. In a controlled feeding trial, we used African catfish (*Clarias gariepinus*), which are one of the most efficient warm water species and became increasingly important in global aquaculture production [36,37]. To avoid an interaction between supplements and nutrients on the microbiome, two isoenergetic experimental diets with different protein sources were utilized. The control diet was based on fishmeal (FM100), and, in the experimental diet, the fishmeal was completely exchanged to spirulina (SP100). We hypothesized that we would find a shift in the microbial community structure. Therefore, we investigated the impact of the algal component in the diet of African catfish on the microbiome, utilizing 16S rRNA amplicon sequencing and based on a previous study on product quality and fatty acid composition [38] (data not yet published). 

## 2. Materials and Methods

### 2.1. Fish and Rearing Conditions

A total of 120 African catfish (*Clarias gariepinus*) full siblings with an average size of 50.1 ± 2.95 g were fed for ten weeks on two different experimental diets (Table 1 and Table 2) [39]. Both groups were fed pelleted 3 mm feed, with the control group (FM100) consisting of 20% fish meal. In the treatment group (SP100), the fish meal was completely replaced by an *Arthrospira platensis* meal. Each treatment was run in triplicate and consisted of 20 fish, which were kept in a recirculated aquaculture system comprising 200 L aquariums with 10 h of dim light and 14 h of darkness. The daily amount of feed consisted of 2% of the fish biomass and was applied in two portions. In order to maintain a constant stocking density, dead fish were removed and replaced with another full sibling of the same weight (± 5 g) and marked with a PIT tag. Replaced fish were excluded from further microbiome analysis. The body weight of the fish was regularly measured, and feed rations were adjusted accordingly. The temperature and the oxygen saturation/content were recorded daily for the recirculation system by a Pond Master sensor (OxyGuard, Farum, Denmark). Over the period of the study, the mean water temperature was 27.0 ± 0.43 °C, while mean oxygen content was 9.9 ± 0.41 mg/L. The pH value was measured weekly by a color scale for indicator solution UNISOL 410 (MACHEREY-NAGEL, Düren, Germany). A NANOCOLOR 300 D (MACHEREY-NAGEL, Düren, Germany) was used to measure ammonium and nitrate contents photometrically. The mean pH of the water was 6.9 ± 0.23, with 0.05–0.12 mg/L ammonium and 0.12–0.26 mg/L nitrate. Both groups were fed pelleted 3 mm feed, with the control group (FM100) consisting of 20% fish meal. In the treatment group (SP100), the fish meal was completely replaced by an *Arthrospira platensis* meal. Each treatment was run in triplicate and consisted of 20 fish, which were kept in a recirculated aquaculture system comprising 200 L aquariums with 10 h of dim light and 14 h of darkness. The daily amount of feed consisted of 2% of the fish biomass and was applied in two portions. In order to maintain a constant stocking density, dead fish were removed and replaced with another full sibling of the same weight (±5 g) and marked with a PIT tag. Replaced fish were excluded from further microbiome analysis. The body weight of the fish was regularly measured, and feed rations were adjusted accordingly. The temperature and the oxygen saturation/content were recorded daily for the recirculation system by a Pond Master sensor (OxyGuard, Farum, Denmark). Over the period of the study, the mean water temperature was 27.0 ± 0.43 °C, while mean oxygen content was 9.9 ± 0.41 mg/L. The pH value was measured weekly by a color scale for indicator solution UNISOL 410 (MACHEREY-NAGEL, Düren, Germany). A NANOCOLOR 300 D (MACHEREY-NAGEL, Düren, Germany) was used to measure ammonium and nitrate contents photometrically. The mean pH of the water was 6.9 ± 0.23, with 0.05–0.12 mg/L ammonium and 0.12–0.26 mg/L nitrate.

### 2.2. Microbiome

#### 2.2.1. Sampling

The microbiome sampling took place at day 70 of feeding. To ensure that the gastrointestinal tract was equally filled, feed was applied to each tank 4 h (staggered every 30 min) before the sampling. Three fish per tank (9 fish/treatment) were killed by a sharp blow to the head and processed for microbiome analysis. The external surfaces of the fish were cleaned with 99.8% pure ethanol and dissected with sterile syringes and forceps. Thereafter, the lower third of the intestine was removed and squeezed out on a sterile petri dish. In total, 220 mg of feces were put into a 2 mL bead beating tube (Sarstedt AG & Co. KG, Nümbrecht, Germany) and placed on dry ice pellets until further processing the same day.

#### 2.2.2. DNA Extraction

DNA was extracted with the QIAamp^®^ Fast DNA Stool Mini Kit (Qiagen, Venlo, The Netherlands) following the manufacturers protocol with following modifications. After the application of InhibitEX buffer, samples were disrupted by a Bead Ruptor Elite (OMNI International, Kennesaw, GA, USA) with 300 μg 0.1–0.2 mm, 100 μg 0.4–0.6 mm, and three 1.4–1.6 mm ceramic beads (Biolabproducts, Bebensee, Germany) on two cycles for 45 s with 6 m/s and 5 min of rest on ice in between. A total of 45 μL of Proteinase K was used, and 50 μL ATE buffer was placed on the QIAmp spin column membrane, incubated for 5 min and centrifuged for 1 min. The filtrate was then applied again on the same QIAmp membrane (to maximize the microbial DNA output) and repeated. The DNA yield was quantified by an Infinite^®^ 200 Pro (TECAN Group Ltd., Männedorf, Switzerland).

#### 2.2.3. 16S rRNA Gene Amplification and Sequencing

In order to amplify 16S rRNA sequences, we used 16S rRNA with the bacterial gene primer pairs S-D-Bact-0341-b-S-17: 5′-CCTACGGGNGGCWGCAG-3′ and S-D-Bact-0785-a-A-21: 5′-GACTACHVGGGTATCTAATCC-3′ targeting the V3–V4 region [40] by using the Phusion High-Fidelity DNA Polymerase (Thermo Fisher Scientific, Schwerte, Germany). The reaction (50 µL) contained 10 µL of 5× Phusion GC buffer, 0.2 µL 50 mM MgCl_2_ solution, 2.5 µL DMSO, 200 µM of each of the four deoxynucleoside triphosphates, and 1 U of Phusion DNA Polymerase. We used 20–30 ng of DNA and 1 µL cDNA per reaction. The PCR reaction was started by an initial denaturation at 98 °C for 1 min, followed by 25 cycles of denaturation at 98 °C for 45 s, annealing at 60 °C for 45 s, and elongation at 72 °C for 30 s. The final elongation was done at 72 °C for 5 min. Each PCR was performed in triplicate so as to reduce any PCR bias. The PCR products were visualized on a 1% agarose gel at 100 V for 1 h to check for bacterial DNA amplicons. The presence of these was then verified with the identification of a PCR product at ~550 bp. These amplicons were then purified using the MagSi-NGS PREP Plus magnetic beads (AMS Biotechnology, Abingdon, UK) with 30 µL bead solution on 25 µL amplicon solution and an elution volume of 30 µL EB buffer. Purified amplicons were sequenced with an Illumina MiSeq and Nextera XT DNA Library Prep Kit chemistry (Illumina, San Diego, CA, USA), resulting in paired-end reads of 2 × 300 bp length [39] by using the Phusion High-Fidelity DNA Polymerase (Thermo Fisher Scientific, Schwerte, Germany). The reaction (50 µL) contained 10 µL of 5× Phusion GC buffer, 0.2 µL 50 mM MgCl_2_ solution, 2.5 µL DMSO, 200 µM of each of the four deoxynucleoside triphosphates, and 1 U of Phusion DNA Polymerase. We used 20–30 ng of DNA and 1 µL cDNA per reaction. The PCR reaction was started by an initial denaturation at 98 °C for 1 min, followed by 25 cycles of denaturation at 98 °C for 45 s, annealing at 60 °C for 45 s, and elongation at 72 °C for 30 s. The final elongation was done at 72 °C for 5 min. Each PCR was performed in triplicate so as to reduce any PCR bias. The PCR products were visualized on a 1% agarose gel at 100 V for 1 h to check for bacterial DNA amplicons. The presence of these was then verified with the identification of a PCR product at ~550 bp. These amplicons were then purified using the MagSi-NGS PREP Plus magnetic beads (AMS Biotechnology, Abingdon, UK) with 30 µL bead solution on 25 µL amplicon solution and an elution volume of 30 µL EB buffer. Purified amplicons were sequenced with an Illumina MiSeq and Nextera XT DNA Library Prep Kit chemistry (Illumina, San Diego, CA, USA), resulting in paired-end reads of 2 × 300 bp length.

#### 2.2.4. Sequence Processing and Analyses

Amplicon sequencing was performed by the Göttingen Genomics Laboratory using the CASAVA software (Illumina, San Diego, CA, USA) for demultiplexing and clipping of adapter sequences from the raw amplicon sequences. Quality filtering was performed using fastp (v0.20.0) [41], and sequences with a phred score of ≥ 20 and a length of ≥ 50 bp were excluded. Soft clipping of low quality base pairs took place with phred score of 20 and a sliding window size of four bases as well as a read correction by overlap and adapter of Illumina Nextera primers. These quality-filtered reads were then merged with the paired end read merger (PEAR v.0.9.11) with default settings [42]. The program cutadapt (v2.5) was used with default settings to remove forward and reverse primer sequences [43]. Subsequently, sequences were then size filtered (≤300 bp were removed) and dereplicated by vsearch (version 2.14.1) [44]. The vsearch module UNOISE3 (default settings) was used for denoising reads with a minimum size of eight reads, and the UCHIME module further excluded chimeric sequences (including de novo chimera and reference-based chimera) against the SILVA SSU 138 NR database [45,46]. The resulting amplicon sequence variants were clustered at 97% by vsearch. OTUs were taxonomically assigned with BLASTn (version 2.9.0) against the SILVA SSU 138 NR database with an identity threshold of 90%. Uncertain blast hits were marked by using identity and query coverage. Additionally, taxonomic assignments for blast hits with (pident + qcovs)/2 ≤ 93% were removed (recommended by SILVA ribosomal RNA database project).

### 2.3. Data Processing

Analysis was carried out with R (version 3.6.3) [47]. Microbiome data were normalized per sample and processed with R package “phyloseq”, and a PcoA plot was computed on phylum level [48]. Alpha diversity was calculated for common metrics (Chao1, ACE, Shannon, Simpson, and Fischer) and a Wilcoxon rank test was performed to compare the observed diversity in FM100 and SP100. Bar plots were produced with “ggplot2” [49].

### 2.4. Sequence Data Deposition

The 16 S rRNA gene amplicon sequences were submitted to the NCBI Sequence Read Archive4 (SRA) under the NCBI BioProject accession number PRJNA723703.

## 3. Results

Bacterial DNA was isolated from 17 of 18 samples. One sample from the FM100 group indicated poor PCR amplification and was excluded from the study. Overall, the microbial community could be classified to 8 phyla, 10 classes, 25 orders, 40 families, 49 genera, and 69 species.

**Figure 1:**
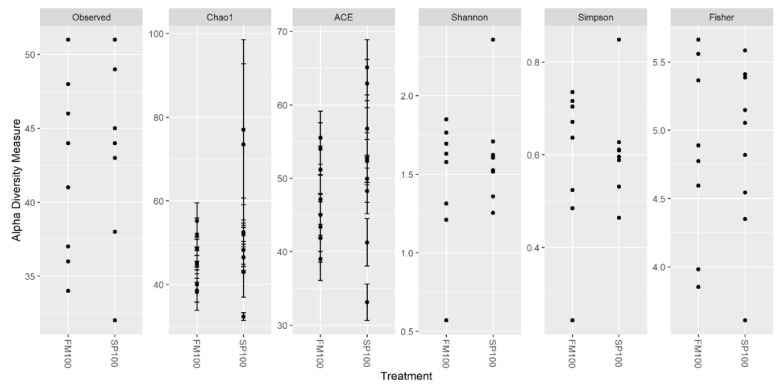
Alpha diversity metrics of microbiome community of African catfish fed with FM100 (n = 8) and SP100 (n = 9) diet. Each point represents one microbiome sample.

Alpha diversity metrics are shown in Figure 1. Between 32–51 bacterial species were observed in the microbiome. Observed richness showed no statistically significant difference (*p* = 0.499). Chao1 and ACE showed two samples of the SP100 group with a notably higher diversity in the FM100 group, but also one sample within this group had the lowest diversity in regard to the FM100 group. Similar findings were represented by the Shannon and the Simpson indices with the lowest diversity in a sample in FM100 and the highest in a sample from SP100. However, Fisher’s alpha parameter seemed to be only slightly different.

**Figure 2:**
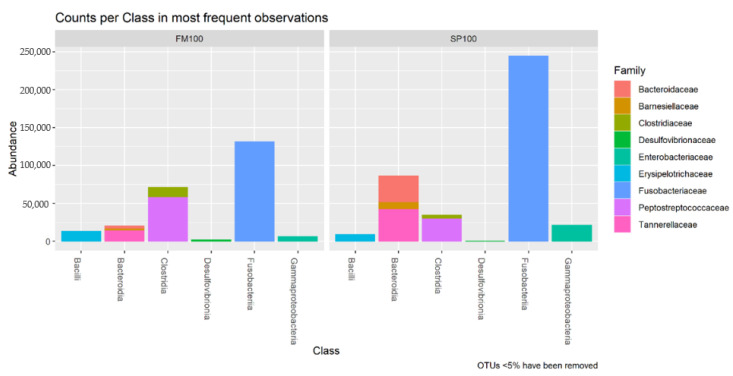
Bacterial counts per class of the most frequent observations (OTUs < 5% were removed) of FM100 (n = 8) and SP100 (n = 9).

Sequencing resulted in 652,898 counts, 248,486 for FM100 and 404,412 for SP100 group. The numbers of counts varied between the samples between 14,419–72,937. We observed the counts per class in the most frequent observations (Figure 2) and the most abundant bacteria on genus level (Table 3). The highest number of counts were dominated by *Fusobacteriia* in FM100 and SP100 with the only taxon from the genus *Cetobacterium*. The second highest counts in FM100 were *Clostridia*, followed by *Bacteroidia*, *Bacilli*, *Gammaproteobacteria*, and *Desulfovibrionia*. In SP100, the second highest counts were observed for *Bacteroidia*, followed by *Clostridia*, *Gammaproteobacteria*, and *Desulfovibrionia*. The class of *Clostridia* was highly abundant in FM100 group, dominated by the genus *Romboutsia*. The family of *Peptostreptococcaceae*, *Bacteroidaceae*, and *Barnesiellaceae* were predominant in *Bacteroidia* and mainly found in spirulina fed fish. Additionally, *Gammaproteobacteria* was mostly abundant in SP100 group and contained primarily the genus *Plesiomonas*.

**Figure 3:**
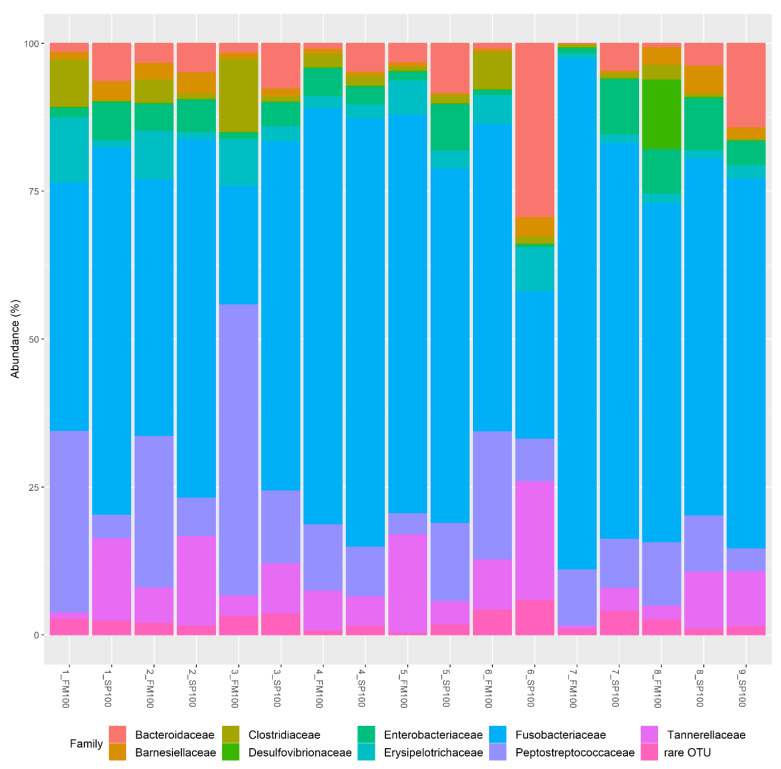
Relative abundance (%) at family level for samples in FM100 (n = 8) and SP100 (n = 9).

**Figure 4:**
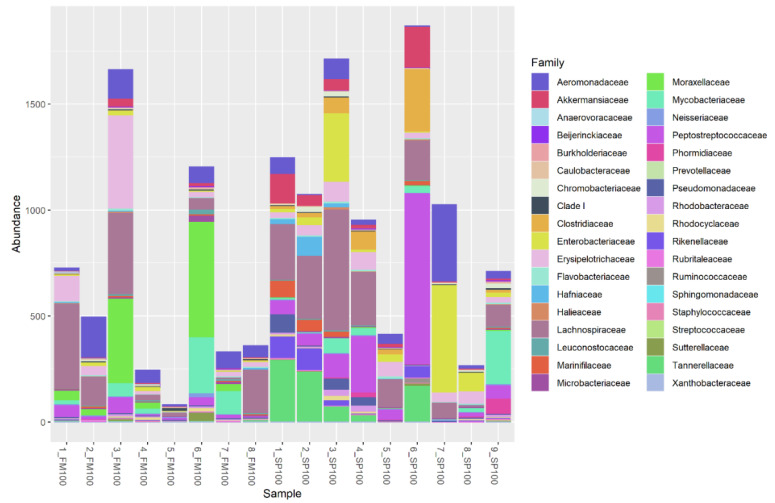
Relative abundance (%) of rare OTUs (<5%) of FM100 (n = 8) and SP100 (n = 9) samples.

Relative abundances for each sample on family level are shown in Figure 3. OTUs (operational taxonomic units) with < 5% were declared as “rare OTUs” (represented in Figure 4). Similar to Figure 2, high percentages of *Fusobacteriia* represented the most abundant bacteria in most of the probes. There were, however, two exceptions to this with 3_FM100 indicating a high proportion of *Peptostreptococcaceae* and 6_SP100 with a high abundancy of *Bacteroidaceae*. As such, overall, we could observe a high variation between samples.

Between 40–53 rare OTUs in FM100 and between 48–58 rare OTUs in SP100 were found to be present in the microbial probes (Figure 4). Rare OTUs were observed in both groups, whereby 6_SP100 showed the highest percentage of rare bacteria in the microbiome. As with the other OTUs, the rare OTUs also showed a high variation between samples.

**Figure 5:**
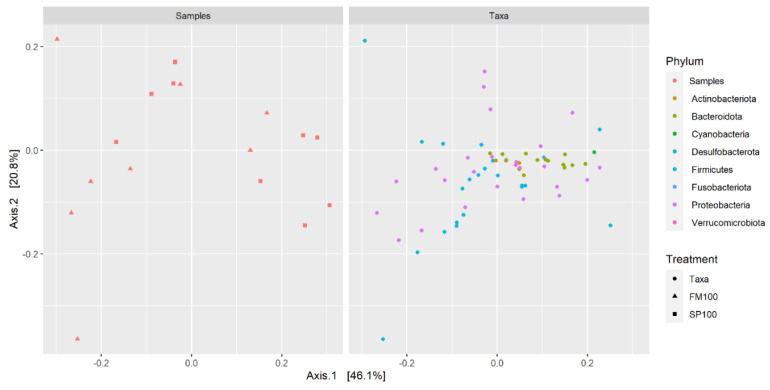
Principal coordinate analysis (PCoA) of FM100 (n = 8) and SP100 (n = 9) samples at phylum level. Each item represents an individual sample.

We used a principal coordinate analysis (PCoA) to confirm previous statistical analysis. The distance matrix was used to detect similarities and differences in microbial community structures (Figure 5). However, neither samples nor taxa were visually distinguishable due to the close clustering. The PCoA showed only low level of variation, as all values were clustered near the origin; therefore, the samples showed only small or no differences. In this case, we were not able to distinguish between dietary groups, and the overall microbiome structure of the gastrointestinal tract was not seen to be affected by the treatment.

## 4. Discussion

Microalgae/cyanobacteria such as spirulina are promising supplements for fishmeal, but information about the impact on the intestinal fish microbiome is highly limited. Only a few studies concentrated on algae supplementation and its effect on the microbiome, but the supplementation levels were rather low. Our study is the first one evaluating a complete microalgae/cyanobacteria supplementation, hoping to find the exclusive effect of spirulina on the microbiome.

In general, the most frequent phyla in fish gut microbiota are *Actinobacteria*, *Bacteroidetes*, *Fusobacteria*, *Firmicutes,* and *Proteobacteria*, which account for over 80% of the total gut bacteria [50], which is also in accordance with our study. We were able to demonstrate that the most abundant microbial family in African catfish was *Fusobacteriia* with the only taxon from the genus *Cetobacterium* in FM100 and SP100 groups. This bacterium is also highly abundant in other fish species such as carp [51], rainbow trout [24], and tilapia [22,28,52]. Both Minich et al. [53] and Bledsoe et al. [54] observed *Cetobacterium somerae* as the most abundant microbial bacteria for African catfish and channel catfish (*Ictalurus punctatus*), respectively. *Cetobacterium somerae* plays an important role in the intestinal microbiome due to its physiological benefits of synthesizing vitamin B-12 and antimicrobial metabolites [55]. The second most abundant genus in our study was *Romboutsia,* which was found to be lower in spirulina-fed fish. This genus is able to utilize carbohydrates and have the capability to ferment numerous amino acids, anaerobic respiration, and metabolic end products in the human intestine [56]. In farmed tilapia (*Oreochromis niloticus*), the abundance of *Romboutsia* is reduced in fish fed with low protein levels [57]. However, both of our experimental diets contained approximately the same percentages of crude protein and essential amino acids. Therefore, we suggest that *Romboutsia* may be unable to degrade the cell wall of microalgae component and, as such, led to the reduced bioavailability of the spirulina protein. Another change on genus level can be seen in the abundance of *Gammaproteobacteria*. The supplementation of microalgae increased the abundance of this genus in fish fed the spirulina-diet. The same effect could be observed with microalgae [22]. In humans, high levels of *Gammaproteobacteria* can be observed in patients with nonalcoholic fatty liver disease [58]. The class of *Gammaproteobacteria* are able to produce endogenous alcohol [59], which may affect liver health. In this case, higher feed intensity and long-term effects of microalgae application may have a negative effect on liver health, but this would require further examination. Within the class of *Gammaproteobacteria*, the genus *Plesiomonas* was more abundant in SP100 than in FM100 samples. *Plesiomonas shigelloides*, which is known to be an aerogenic gram-negative bacteria [60], is often found in tropical climates [61] and in various fish species [54,62,63]. As *P. shigelloides* was proven to have an antimicrobial effect, its presence in gut microbiome may have a positive effect on the overall health of the fish [62]. 

Our study indicates that a full supplementation of fishmeal with spirulina has the potential to alter the diversity of microbiome samples, leading to a higher bacterial diversity in the gut. In this context, we were able to find a possible increase of diversity in different metrics but were not able to find a significant statistical difference in the observed richness. Due to a small number of samples and a high variation between all samples, the overall microbial community structure was not affected. Similar findings were presented by Lyons et al. [24], who also found higher OTU counts and a higher diversity (for Chao index) in rainbow trout (*Oncorhynchus mykiss*) fed with a 5% whole-cell microalgae ingredient (*Schizochytrium limacinum*) for 15 weeks, but they were also unable to observe changes in the microbial community structure. The authors explained this as an effect by polysaccharides contained in the microalga that led to an adaptation of the gut microbiota but did not affect the bacterial community structure. Aside from this, they were also able to observe a strong variation between microbiome samples. Souza et al. [22] determined also an increasing diversity (for Chao index) with a 1.2% *Schizochytrium* sp. supplementation for Nile tilapia (*Oreochromis niloticus*).

It is known that, in mammals, the microbial gut diversity increases from carnivorous to omnivorous to herbivorous [64], and the same occurs in fish [65]. In addition, the gut length also increases from carnivorous to omnivorous to herbivores species [66]. Normally, a long gut favors anaerobic bacteria, which are more common in herbivorous gut microbiome [67]. A slow passage of feed ingredients through the longer digestive tract may increase the time for microbial fermentation, leading to an increase in the microbial diversity [24] and thus associated with an improved dietary digestion [68]. In herbivorous fish, the abundance of cellulolytic bacteria is found to be higher in the gut [65,69,70]. We were able to see this effect due to an increase of some microbial phyla such as *Firmicutes* and *Bacteroidia* in the microbiome of those spirulina-fed. These bacterial members are able to improve cellulose digestion in herbivorous fish [69,71]. As the African catfish is an omnivorous fish with a relatively short gut length, the rate of passage for microalgae components may be too short in the gastrointestinal tract for the fermentation process to occur and to influence the microbiome structure. Furthermore, dietary factors also have the ability to alter the intestinal morphology [72]. Souza et al. [22] did not find an effect on the morphological structure or integrity of the intestinal villi due to a microalgae supplementation for Nile tilapia.

As described earlier, environmental factors can affect the microbiome of fish. To minimize the environmental effect, we comprised all experimental fish with the same water from a recirculation aquaculture system. The water temperature was within the optimum range of 25–28 °C [73], and dissolved oxygen was in an appropriate range [74]. Nitrate and ammonium concentrations were below the upper limit throughout the whole test period [74,75,76]. It cannot be ruled out that the effects of the environmental factors have a stronger shaping effect on the gastrointestinal tract and its microbiome than those of the diet.

## 5. Conclusions

Spirulina is a promising microalgae/cyanobacteria for fish nutrition and can be considered as a suitable alternative for fishmeal. Previous studies on the impact of microalgae on the microbiome were performed using low levels of algae inclusion. Our study is the first one focusing on a total replacement of fishmeal with spirulina and its effect on the microbiome of African catfish. While the data were not statistically significant, there was some indication that the diversity levels could be altered by supplementation; this will need to be further investigated in more depth. A further adaptation of the intestinal microbiome to the supplemented microalgae was seen at the bacterial genus level. We were unable to see an effect on the overall microbiome structure, but further investigation into the effect on alternative fish species could prove more insightful. As manipulation of the microbiome was shown to improve health as well as nutrient utilization in fish, it could therefore lead to further improvements in aquaculture production as a whole.

## Figures and Tables

**Table 1:** Feed ingredients of FM100 and SP100 diets.

Ingredient (% Dry Matter)	FM100	SP100
Fishmeal ^1^	20.00	0.00
Spirulina ^2^	0.00	20.00
Fish oil	10.70	10.70
Wheat meal	14.00	12.50
Wheat gluten	20.00	21.50
Soy protein concentrate ^3^	20.00	20.00
Rapeseed oil	10.70	10.70
Vit./Min. Premix	1.00	1.00
CaHPO_4_	1.00	1.00
CMC (Binder)	1.29	1.08
TiO_2_ (Marker)	0.50	0.50
Fe_3_O_4_–black (Dye)	0.07	0.07
L-Lysin (HCL-Lys, 78% Lys)	0.70	0.90
D,L-Methionine	0.01	0.04
L-Tryptophan	0.03	0.01

^1^ Crude protein: 62% as is, ^2^ Crude protein: 63% as is, ^3^ Crude Protein: 67% as is.

**Table 2:** Approximate composition (% fresh matter) of FM100 and SP100 diets.

Approximate Composition (%)	FM100	SP100
Dry matter	94.6	94.0
Crude protein (N × 6.25)	45.4	45.7
Crude lipids	24.6	23.9
N-free extracts	17.5	19.0
Crude ash	7.1	5.4
Gross energy [MJ/kg]	23.4	23.5
Digestible energy [MJ/kg]	20.0	20.0
Essential amino acids ^1^	26.6	26.8

^1^ % in feed dry matter.

**Table 3:** Mean values ± SD of percentual abundant of bacteria on genus level in FM100 and SP100.

Genus	FM100	SP100
***Cetobacterium***	54.98 ± 20.43	58.90 ± 13.34
***Romboutsia***	20.30 ± 14.88	8.14 ± 3.26
***Macellibacteroides***	4.15 ± 4.10	5.05 ± 2.50
***Plesiomonas***	2.95 ± 2.47	5.66 ± 2.95
***Bacteroides***	1.50 ± 0.61	8.82 ± 5.72
***[Anaerorhabdus] furcosa group***	3.44 ± 2.96	1.88 ± 1.92
***[Barnesiellaceae]* uncultured**	1.30 ± 1.15	2.17 ± 1.72
***Clostridium sensu stricto 1***	2.39 ± 1.94	0.72 ± 0.38
***Turicibacter***	2.25 ± 2.30	0.21 ± 0.19

## Data Availability

The datasets generated for this study are available on NCBI BioProject accession number: PRJNA723703.

## References

[1] FAO (2016). State of World Fisheries and Aquaculture 2020: Sustainability in Action.

[2] World Bank (2013). Fish to 2030: Prospects for Fisheries and Aquaculture.

[3] García S.M. (2003). The Ecosystem Approach to Fisheries: Issues, Terminology, Principles, Institutional Foundations, Implementation and Outlook.

[4] Becker E.W. (2007). Micro-algae as a source of protein. Biotechnol. Adv..

[5] Rana K.J., Siriwardena S., Hasan M.R. (2009). Impact of Rising Feed Ingredient Prices on Aquafeeds and Aquaculture Production.

[6] Ytrestøyl T., Aas T.S., Åsgård T. (2015). Utilisation of feed resources in production of Atlantic salmon (Salmo salar) in Norway. Aquaculture.

[7] Ragaza J.A., Hossain M.S., Meiler K.A., Velasquez S.F., Kumar V. (2020). A review on Spirulina: Alternative media for cultivation and nutritive value as an aquafeed. Rev. Aquac..

[8] Yarnold J., Karan H., Oey M., Hankamer B. (2019). Microalgal Aquafeeds As Part of a Circular Bioeconomy. Trends Plant Sci..

[9] Dernekbasi S., Una H., Karayucel I., Aral O. (2010). Effect of Dietary Supplementation of Different Rates of Spirulina (Spirulina platensis) on Growth and Feed Conversion in Guppy (Poecilia reticulata Peters, 1860). J. Anim. Vet. Adv..

[10] Raji A.A., Alaba P.A., Yusuf H., Abu Bakar N.H., Mohd Taufek N., Muin H., Alias Z., Milow P., Abdul Razak S. (2018). Fishmeal replacement with Spirulina Platensis and Chlorella vulgaris in African catfish (Clarias gariepinus) diet: Effect on antioxidant enzyme activities and haematological parameters. Res. Vet. Sci..

[11] Promya J., Chitmanat C. (2011). The effects of Spirulina platensis and Cladophora Algae on the Growth Performance, Meat Quality and Immunity Stimulating Capacity of the African Sharptooth Catfish (Clarias gariepinus). Int. J. Agric. Biol..

[12] Jafari S.M.A., Rabbani M., Emtyazjoo M., Piryaei F. (2014). Effect of dietary Spirulina platensis on fatty acid composition of rainbow trout (Oncorhynchus mykiss) fillet. Aquac. Int..

[13] Roohani A.M., Abedian Kenari A., Fallahi Kapoorchali M., Borani M.S., Zoriezahra S.J., Smiley A.H., Esmaeili M., Rombenso A.N. (2019). Effect of spirulina Spirulina platensis as a complementary ingredient to reduce dietary fish meal on the growth performance, whole-body composition, fatty acid and amino acid profiles, and pigmentation of Caspian brown trout (Salmo trutta caspius) juveniles. Aquac. Nutr..

[14] Teimouri M., Yeganeh S., Amirkolaie A.K. (2016). The effects of Spirulina platensis meal on proximate composition, fatty acid profile and lipid peroxidation of rainbow trout (Oncorhynchus mykiss) muscle. Aquac. Nutr..

[15] Desai A.R., Links M.G., Collins S.A., Mansfield G.S., Drew M.D., van Kessel A.G., Hill J.E. (2012). Effects of plant-based diets on the distal gut microbiome of rainbow trout (Oncorhynchus mykiss). Aquaculture.

[16] Estruch G., Collado M.C., Peñaranda D.S., Tomás Vidal A., Jover Cerdá M., Pérez Martínez G., Martinez-Llorens S. (2015). Impact of Fishmeal Replacement in Diets for Gilthead Sea Bream (Sparus aurata) on the Gastrointestinal Microbiota Determined by Pyrosequencing the 16S rRNA Gene. PLoS ONE.

[17] Ingerslev H.-C., von Gersdorff Jørgensen L., Lenz Strube M., Larsen N., Dalsgaard I., Boye M., Madsen L. (2014). The development of the gut microbiota in rainbow trout (Oncorhynchus mykiss) is affected by first feeding and diet type. Aquaculture.

[18] Ringø E., Sperstad S., Myklebust R., Refstie S., Krogdahl Å. (2006). Characterisation of the microbiota associated with intestine of Atlantic cod (Gadus morhua L.). Aquaculture.

[19] Schmidt V., Amaral-Zettler L., Davidson J., Summerfelt S., Good C. (2016). Influence of Fishmeal-Free Diets on Microbial Communities in Atlantic Salmon (Salmo salar) Recirculation Aquaculture Systems. Appl. Environ. Microbiol..

[20] Xia J.H., Lin G., Fu G.H., Wan Z.Y., Lee M., Wang L., Liu X.J., Yue G.H. (2014). The intestinal microbiome of fish under starvation. BMC Genom..

[21] Zarkasi K.Z., Taylor R.S., Abell G.C.J., Tamplin M.L., Glencross B.D., Bowman J.P. (2016). Atlantic Salmon (Salmo salar L.) Gastrointestinal Microbial Community Dynamics in Relation to Digesta Properties and Diet. Microb. Ecol..

[22] de Souza F.P., de Lima E.C.S., Urrea-Rojas A.M., Suphoronski S.A., Facimoto C.T., da Silva Bezerra Júnior J., de Oliveira T.E.S., de Pádua Pereira U., Di Santis G.W., de Oliveira C.A.L. (2020). Effects of dietary supplementation with a microalga (Schizochytrium sp.) on the hemato-immunological, and intestinal histological parameters and gut microbiota of Nile tilapia in net cages. PLoS ONE.

[23] Cerezuela R., Fumanal M., Tapia-Paniagua S.T., Meseguer J., Moriñigo M.A., Esteban M.A. (2012). Histological alterations and microbial ecology of the intestine in gilthead seabream (Sparus aurata L.) fed dietary probiotics and microalgae. Cell Tissue Res..

[24] Lyons P.P., Turnbull J.F., Dawson K.A., Crumlish M. (2017). Effects of low-level dietary microalgae supplementation on the distal intestinal microbiome of farmed rainbow trout Oncorhynchus mykiss (Walbaum). Aquac. Res..

[25] Sullam K.E., Essinger S.D., Lozupone C.A., O’Connor M.P., Rosen G.L., Knight R., Kilham S.S., Russell J.A. (2012). Environmental and ecological factors that shape the gut bacterial communities of fish: A meta-analysis. Mol. Ecol..

[26] Roeselers G., Mittge E.K., Stephens W.Z., Parichy D.M., Cavanaugh C.M., Guillemin K., Rawls J.F. (2011). Evidence for a core gut microbiota in the zebrafish. ISME J..

[27] Schmidt V.T., Smith K.F., Melvin D.W., Amaral-Zettler L.A. (2015). Community assembly of a euryhaline fish microbiome during salinity acclimation. Mol. Ecol..

[28] Zhang M., Sun Y., Liu Y., Qiao F., Chen L., Liu W.-T., Du Z., Li E. (2016). Response of gut microbiota to salinity change in two euryhaline aquatic animals with reverse salinity preference. Aquaculture.

[29] Ray C. (2016). Characterization of the Gut and Skin Microbiomes of Wild-Caught Fishes from Lake Guntersville, , Alabama. Master’s Thesis.

[30] Zarkasi K.Z., Abell G.C.J., Taylor R.S., Neuman C., Hatje E., Tamplin M.L., Katouli M., Bowman J.P. (2014). Pyrosequencing-based characterization of gastrointestinal bacteria of Atlantic salmon (Salmo salar L.) within a commercial mariculture system. J. Appl. Microbiol..

[31] Smith C.C.R., Snowberg L.K., Gregory Caporaso J., Knight R., Bolnick D.I. (2015). Dietary input of microbes and host genetic variation shape among-population differences in stickleback gut microbiota. ISME J..

[32] Ye L., Amberg J., Chapman D., Gaikowski M., Liu W.-T. (2014). Fish gut microbiota analysis differentiates physiology and behavior of invasive Asian carp and indigenous American fish. ISME J..

[33] Ringø E., Zhou Z., Vecino J., Wadsworth S., Romero J., Krogdahl Å., Olsen R.E., Dimitroglou A., Foey A., Davies S. (2016). Effect of dietary components on the gut microbiota of aquatic animals. A never-ending story?. Aquac. Nutr..

[34] Romero J., Ringø E., Merrifield D.L., Merrifield D., Ringø E. (2014). The Gut Microbiota of Fish. Aquaculture Nutrition: Gut Health, Probiotics, and Prebiotics.

[35] Llewellyn M.S., Boutin S., Hoseinifar S.H., Derome N. (2014). Teleost microbiomes: The state of the art in their characterization, manipulation and importance in aquaculture and fisheries. Front. Microbiol..

[36] Palm H.W., Knaus U., Wasenitz B., Bischoff A.A., Strauch S.M. (2018). Proportional up scaling of African catfish (Clarias gariepinus Burchell, 1822) commercial recirculating aquaculture systems disproportionally affects nutrient dynamics. Aquaculture.

[37] Bovendeur J., Eding E.H., Henken A.M. (1987). Design and performance of a water recirculation system for high-density culture of the African catfish, Clarias gariepinus (Burchell 1822). Aquaculture.

[38] Rosenau S., Oertel E., Dietz C., Wessels S., Tetens J., Mörlein D., Ciulu M. Total Replacement of Fishmeal by Spirulina (Arthrospira platensis) and its Effect on Growth Performance and Product Quality of African catfish (Clarias gariepinus). Sustainability.

[39] Dietz C., Sünder A., Liebert F. (2020). Does Genetic Background of Rainbow Trout Impact on Growth and Feed Utilization Following Fishmeal Substitution by Partly Defatted Insect Meal (Hermetia illucens) or Microalgae Powder (Arthrospira platensis)?: 74rd Conference 3rd–5th March 2020 in Göttingen.

[40] Klindworth A., Pruesse E., Schweer T., Peplies J., Quast C., Horn M., Glöckner F.O. (2013). Evaluation of general 16S ribosomal RNA gene PCR primers for classical and next-generation sequencing-based diversity studies. Nucleic Acids Res..

[41] Chen S., Zhou Y., Chen Y., Gu J. (2018). fastp: An ultra-fast all-in-one FASTQ preprocessor. Bioinformatics.

[42] Zhang J., Kobert K., Flouri T., Stamatakis A. (2014). PEAR: A fast and accurate Illumina Paired-End reAd mergeR. Bioinformatics.

[43] Martin M. (2011). Cutadapt removes adapter sequences from high-throughput sequencing reads. EMBnet J..

[44] Rognes T., Flouri T., Nichols B., Quince C., Mahé F. (2016). VSEARCH: A versatile open source tool for metagenomics. PeerJ.

[45] Bolyen E., Rideout J.R., Dillon M.R., Bokulich N.A., Abnet C.C., Al-Ghalith G.A., Alexander H., Alm E.J., Arumugam M., Asnicar F. (2019). Reproducible, interactive, scalable and extensible microbiome data science using QIIME 2. Nat. Biotechnol..

[46] Quast C., Pruesse E., Yilmaz P., Gerken J., Schweer T., Yarza P., Peplies J., Glöckner F.O. (2013). The SILVA ribosomal RNA gene database project: Improved data processing and web-based tools. Nucleic Acids Res..

[47] R Core Team (2020). R: A Language and Environment for Statistical Computing.

[48] McMurdie P.J., Holmes S. (2013). phyloseq: An R package for reproducible interactive analysis and graphics of microbiome census data. PLoS ONE.

[49] Wickham H. (2016). Ggplot2: Elegant Graphics for Data Analysis.

[50] Yukgehnaish K., Kumar P., Sivachandran P., Marimuthu K., Arshad A., Paray B.A., Arockiaraj J. (2020). Gut microbiota metagenomics in aquaculture: Factors influencing gut microbiome and its physiological role in fish. Rev. Aquac..

[51] Eichmiller J.J., Hamilton M.J., Staley C., Sadowsky M.J., Sorensen P.W. (2016). Environment shapes the fecal microbiome of invasive carp species. Microbiome.

[52] Fan L., Chen J., Meng S., Song C., Qiu L., Hu G., Xu P. (2017). Characterization of microbial communities in intensive GIFT tilapia (Oreochromis niloticus) pond systems during the peak period of breeding. Aquac. Res..

[53] Minich J.J., Zhu Q., Xu Z.Z., Amir A., Ngochera M., Simwaka M., Allen E.E., Zidana H., Knight R. (2018). Microbial effects of livestock manure fertilization on freshwater aquaculture ponds rearing tilapia (Oreochromis shiranus) and North African catfish (Clarias gariepinus). Microbiologyopen.

[54] Bledsoe J.W., Peterson B.C., Swanson K.S., Small B.C. (2016). Ontogenetic Characterization of the Intestinal Microbiota of Channel Catfish through 16S rRNA Gene Sequencing Reveals Insights on Temporal Shifts and the Influence of Environmental Microbes. PLoS ONE.

[55] Tsuchiya C., Sakata T., Sugita H. (2008). Novel ecological niche of Cetobacterium somerae, an anaerobic bacterium in the intestinal tracts of freshwater fish. Lett. Appl. Microbiol..

[56] Gerritsen J., Hornung B., Ritari J., Paulin L., Rijkers G.T., Schaap P.J., de Vos W.M., Smidt H. (2019). A Comparative and Functional Genomics Analysis of the Genus Romboutsia Provides Insight into Adaptation to an Intestinal Lifestyle. bioRxiv.

[57] Zhu H.-J., Qiang J., Tao Y.-F., Ngoepe T.K., Bao J.-W., Chen D.-J., Xu P. (2020). Physiological and gut microbiome changes associated with low dietary protein level in genetically improved farmed tilapia (GIFT, Oreochromis niloticus) determined by 16S rRNA sequence analysis. Microbiologyopen.

[58] Michail S., Lin M., Frey M.R., Fanter R., Paliy O., Hilbush B., Reo N.V. (2015). Altered gut microbial energy and metabolism in children with non-alcoholic fatty liver disease. FEMS Microbiol. Ecol..

[59] Ren N., Xing D., Rittmann B.E., Zhao L., Xie T., Zhao X. (2007). Microbial community structure of ethanol type fermentation in bio-hydrogen production. Environ. Microbiol..

[60] Ahmad M., Aggarwal M., Ahmed A. (1998). Bloody diarrhea caused by Plesiomonas shigelloides proctitis in a human immunodeficiency virus-infected patient. Clin. Infect. Dis..

[61] Brenden R.A., Miller M.A., Janda M.J. (1988). Clinical Disease Spectrum and Pathogenic Factors Associated with Plesiomonas shigelloides Infections in Humans. Rev. Infect. Dis..

[62] Sugita H., Shibuya K., Shimooka H., Deguchi Y. (1996). Antibacterial abilities of intestinal bacteria in freshwater cultured fish. Aquaculture.

[63] Suphoronski S.A., Chideroli R.T., Facimoto C.T., Mainardi R.M., Souza F.P., Lopera-Barrero N.M., Jesus G.F.A., Martins M.L., Di Santis G.W., de Oliveira A. (2019). Effects of a phytogenic, alone and associated with potassium diformate, on tilapia growth, immunity, gut microbiome and resistance against francisellosis. Sci. Rep..

[64] Ley R.E., Hamady M., Lozupone C., Turnbaugh P.J., Ramey R.R., Bircher J.S., Schlegel M.L., Tucker T.A., Schrenzel M.D., Knight R. (2008). Evolution of mammals and their gut microbes. Science.

[65] Li H., Wu S., Wirth S., Hao Y., Wang W., Zou H., Li W., Wang G. (2016). Diversity and activity of cellulolytic bacteria, isolated from the gut contents of grass carp (Ctenopharyngodon idellus) (Valenciennes) fed on Sudan grass (Sorghum sudanense) or artificial feedstuffs. Aquac. Res..

[66] Karachle P.K., Stergiou K.I. (2010). Intestine Morphometrics of Fishes: A Compilation and Analysis of Bibliographic Data. Acta Ichthyol. Piscat..

[67] Escalas A., Auguet J.-C., Avouac A., Seguin R., Gradel A., Borrossi L., Villéger S. (2021). Ecological Specialization Within a Carnivorous Fish Family Is Supported by a Herbivorous Microbiome Shaped by a Combination of Gut Traits and Specific Diet. Front. Mar. Sci..

[68] Bäckhed F., Ley R.E., Sonnenburg J.L., Peterson D.A., Gordon J.I. (2005). Host-bacterial mutualism in the human intestine. Science.

[69] Wu S., Wang G., Angert E.R., Wang W., Li W., Zou H. (2012). Composition, diversity, and origin of the bacterial community in grass carp intestine. PLoS ONE.

[70] Li H., Zheng Z., Cong-xin X., Bo H., Chao-yuan W., Gang H., Noakes D.L.G., Romero A., Zhao Y., Zhou Y. (2009). Isolation of cellulose—producing microbes from the intestine of grass carp (Ctenopharyngodon idellus). Chinese Fishes.

[71] Nayak S.K. (2010). Role of gastrointestinal microbiota in fish. Aquac. Res..

[72] Santigosa E., Sánchez J., Médale F., Kaushik S., Pérez-Sánchez J., Gallardo M.A. (2008). Modifications of digestive enzymes in trout (Oncorhynchus mykiss) and sea bream (Sparus aurata) in response to dietary fish meal replacement by plant protein sources. Aquaculture.

[73] Ogunji J.O., Awoke J. (2017). Effect of environmental regulated water temperature variations on survival, growth performance and haematology of African catfish, Clarias gariepinus. Our Nat..

[74] Peteri A., Nandi S., Chowdhury S.N. (1992). Manual on Seed Production of African Catfish (Clarias gariepinus).

[75] Schram E., Roques J.A.C., Abbink W., Yokohama Y., Spanings T., de Vries P., Bierman S., van de Vis H., Flik G. (2014). The impact of elevated water nitrate concentration on physiology, growth and feed intake of African catfish Clarias gariepinus (Burchell 1822). Aquac. Res..

[76] Schram E., Roques J.A., Abbink W., Spanings T., de Vries P., Bierman S., de van Vis H., Flik G. (2010). The impact of elevated water ammonia concentration on physiology, growth and feed intake of African catfish (Clarias gariepinus). Aquaculture.

